# Giant Appendiceal Mucocele with High Grade Mucinous Neoplasm—Case Report and Review of the Literature

**DOI:** 10.3390/diagnostics14212429

**Published:** 2024-10-30

**Authors:** Laurentiu Vasile Sima, Cristina Ana-Maria Dan, Flavia Zara, Raluca Maria Closca, Alexandra Christa Sima, Cristina Oana Saracin, Radu Gheorghe Dan, Diana Maria Orzata

**Affiliations:** 1 Department of Surgery I, University of Medicine and Pharmacy “Victor Babes”, 300041 Timisoara, Romania; sima.laurentiu@umft.ro (L.V.S.); cristina.dan@umft.ro (C.A.-M.D.); 2 Department of Microscopic Morphology, University of Medicine and Pharmacy “Victor Babes”, 300041 Timisoara, Romania; flavia.zara@umft.ro (F.Z.); raluca.moaca@umft.ro (R.M.C.); 3 Department of Pathology, Emergency City Hospital, 300254 Timisoara, Romania; 4 Department of Internal Medicine II, University of Medicine and Pharmacy “Victor Babes”, 300041 Timisoara, Romania; sima.alexandra@umft.ro; 5 Department of Radiology, Emergency City Hospital, 300254 Timisoara, Romania; oana.cristina.voicu@gmail.com; 6 Department of Emergency General Surgery, Emergency City Hospital, 300254 Timisoara, Romania; dianaorzata@gmail.com

**Keywords:** mucocele, appendicitis, right hemicolectomy, mucinous neoplasm, high grade dysplasia

## Abstract

Appendiceal mucocele is a rare entity first described by Carl von Rokitansky, characterized by cystic dilatation of the appendiceal lumen due to obstruction, epithelial proliferation or inflammation and accumulation of mucoid material. The cause can be either neoplastic or non-neoplastic. Patients with appendiceal mucocele can be asymptomatic or present with right lower quadrant pain which may mimic acute appendicitis. We present the case of a 68-year-old male, who presented to the Emergency Room with a two-day history of right lower quadrant pain, nausea, vomiting and loss of appetite. Abdominal examination revealed tenderness over the Mc Burney point with localized guarding and laboratory results showed leukocytosis with neutrophilia. The abdominal computed tomography revealed a cystic dilated appendix, with a length of 130 mm and a diameter of 75 mm, situated ascending retrocecal and associating peri-appendicular inflammatory changes. The patient underwent right hemicolectomy with side-to-side ileo-colonic anastomosis, due to a wide intraluminal communication between the appendix and the cecum and the inflammation of both structures. Histopathological examination showed both high-grade and low-grade appendiceal mucinous neoplasm.

## 1. Introduction

Appendiceal mucocele is a term that refers to the dilatation of the appendiceal lumen as a result of abnormal mucoid material accumulation [1]. This condition represents 0.3–0.7% of appendiceal pathology and 8% of appendiceal tumors [2] and may appear as a consequence of cystadenoma, cystadenocarcinoma, mucosal hyperplasia or inflammatory/obstructive processes. Most patients are over 45 years of age. While in simple inflammatory or obstructive changes or in mucosal hyperplasia, the appendix may be unremarkable, only slightly dilatated, when it comes to cystadenoma or cystadenocarcinoma, the appearance is of a cystically dilated appendix, reaching a diameter of 6 cm or more, with a fibrous wall. The most common clinical diagnosis is of acute appendicitis, but it can also be an asymptomatic, unsuspected radiological or surgical finding [3]. Clinical symptoms include abdominal pain in the right lower quadrant, a palpable abdominal mass, weight loss, nausea, vomiting and, in about 8% of cases, acute appendicitis [4,5]. On ultrasound, an appendiceal mucocele appears as an ovoid or pear-shaped cystic mass, with internal concentric echogenic layers of mucin known as “onionskin” appearance. On computed tomography (CT), the mucocele of the appendix appears as a tubular structure that is contiguous with the cecum and filled with homogenous, low attenuation content. Proper diagnosis and imaging are crucial for an adequate surgical treatment [6].

According to the histological characteristics of the intraluminal obstruction, appendiceal mucoceles are classified in 4 categories: inflammatory/obstructive mucocele, mucosal hyperplasia, mucinous cystadenoma and mucinous cystadenocarcinoma.

Mucinous cystadenoma and cystadenocarcinoma are the main histological types of appendiceal neoplasms. The aspect is of a voluminous cystically dilated appendix, reaching a diameter of more than 6 cm, with fibrous but thin walls, associated with inflammatory changes, increased intraluminal pressure and dilated vessels that predispose to wall penetration of the mucinous cells or to wall ulceration and consecutive peritoneal dissemination of the mucoid material, known as pseudomyxoma peritonei [3,7]. Based on the degree of mucosal involvement, they are classified as low-grade (LAMN) appendiceal mucinous neoplasms represented by mucinous cystadenoma or borderline neoplasm of the appendix and high-grade mucinous (HAMN) tumors represented by mucinous cystadenocarcinoma. Low-grade appendiceal mucinous neoplasms are well-differentiated lesions with a slow-growing pattern, being quite similar to adenoma. The appendiceal wall is usually fibrotic and may contain calcification as signs of chronic lesion. The high-grade appendiceal neoplasms invade beyond the muscularis mucosa and are characterized by a destructive pattern of growth. Considering the uninterrupted accumulation of mucus and the constant increase in the intraluminal pressure, both histological types are prone to perforation and dissemination into the peritoneal cavity, leading to the occurrence of pseudomyxoma peritonei [8,9]. HAMN has a far higher risk of recurrence then LAMN, leading the American Joint Committee on Cancer (AJCC) to recommend staging HAMN using the same scheme as for invasive appendiceal adenocarcinoma [10].

We present the case of a 68-year-old male patient who was admitted to our hospital with signs of acute appendicitis. The CT examination revealed a giant appendiceal mucocele and a right colectomy with ileo-colonic anastomosis was performed. The microscopic examination of the harvested specimen showed both low-grade and high-grade mucinous neoplasm.

## 2. Case Report

A 68-year-old man presents in the emergency room, complaining of pain in the right lower quadrant, nausea, vomiting and loss of appetite, symptoms that started 2 days ago and were preceded by low grade fever (37.5 °C) associated with chills. The patient is known to have high blood pressure treated with specific medication and no other diseases. History taking reveals that he underwent a routine abdominal ultrasound investigation 3 months ago that showed a large cystic mass in the right iliac fossa. In order to elucidate the nature of this mass, a contrast-enhanced CT of the abdomen and the pelvis was performed.

**Figure 1:**
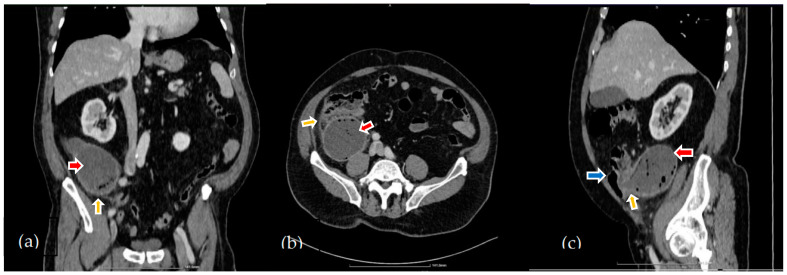
The CT findings on admission—contrast enhanced CT, venous phase, multiplanar reconstruction—coronal, axial and sagital (**a**–**c**): (**a**) increased size of the appendix (red arrow) and inflammatory changes in the peri-appendicular space with fat stranding (orange arrow); (**b**) fluid content and air inclusions in the enlarged appendicular lumen (red arrow), peri-appendicular fat stranding (orange arrow); (**c**) thickening of the appendix walls predominantly at the base (orange arrow) and ascending retrocecal positioning of the appendix (cecum-blue arrow, appendix-red arrow).

On clinical examination, the abdomen is painful on palpation in the right lower quadrant, with localized muscle guarding and a positive Blumberg sign. Laboratory tests reveal the presence of elevated leukocyte count (17,500/mm^3^, with a normal range of 4000–10,000/mm^3^) associated with neutrophilia (14,330/mm^3^, normal range: 2000–7000/mm^3^). There was a slight increase in the procalcitonin level (0.16 ng/mL, normal range < 0.05 ng/mL). According to all these findings, the initial suspicion is of acute appendicitis. A contrast-enhanced CT of the abdomen and the pelvis is performed, showing an appendix with the base near the right antero-superior iliac spine, situated behind the cecum, with an ascending tract, having a length of 13 cm and a markedly enlarged pseudocystic lumen, with a diameter of 7.5 cm. At the implantation site in the cecum, the wall is significantly thickened; the other parts of the wall are slightly thickened and show inflammation. In the lumen, fluid and air inclusions are visualized. Inflammatory changes are also present in the peri-appendicular space and towards the one-sided parietal-colic space, mild fluid infiltrations being detected (Figure 1a–c).

**Figure 2:**
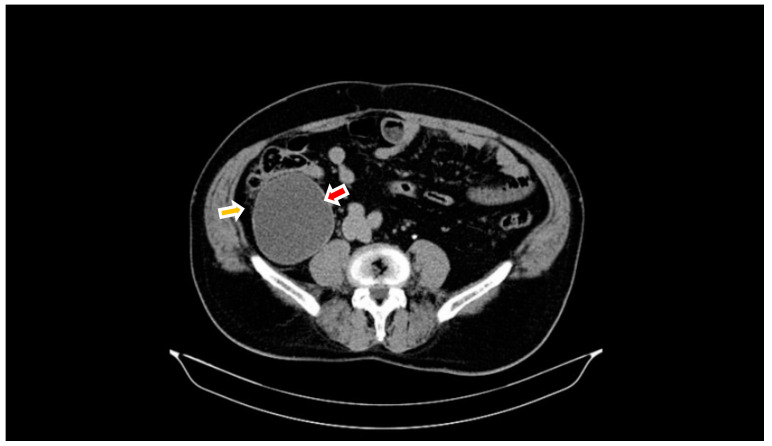
The CT findings 3 months before admission—venous phase CT: increased size of the appendix (red arrow) and the absence of peri-appendicular and pericecal inflammatory changes (orange arrow).

The comparison of the 2 different CT scans showed that the general aspect is quite similar, but the peri-appendicular and pericecal inflammation was not present 3 months ago (Figure 2).

We decided not to perform a colonoscopy, considering it a risky investigation due to the inflammation of the appendix and the cecum. More than that, the therapeutic strategy did not depend on the result of the colonoscopy.

The patient was admitted to the surgery department and the surgical intervention was prepared by starting antibiotics, intravenous fluids and electrolytes. During surgery, the appendix was found to be significantly enlarged, having a diverticular aspect with a wide implantation base and a diameter of approximately 10 cm, with a sclerotic but intact wall, without signs of perforation. It was covered with false membranes and was situated behind the cecum, having an ascending tract. The cecum also appeared with sclerotic, slightly thickened walls in the area of the implantation of the appendiceal base; the terminal ileum appeared normal. Considering the very wide implantation base and the wide communication with the lumen of the cecum, the aspect of the wall of the cecum, as well as the suspicion of malignancy, we decided to perform a right hemicolectomy, excising the piece en bloc, with side-to-side hand-sewn ileo-colonic anastomosis. After surgery, antibiotics were continued for 5 days. The patient had a favorable outcome, with resumption of bowel transit, mobilization and initiation of enteral feeding at the second postoperative day. The drainage revealed a chylous fluid, up to 1000 mL/day, with a gradual reduction. The patient was discharged 14 days after surgery, when the abdominal drainage was suppressed.

**Figure 3:**
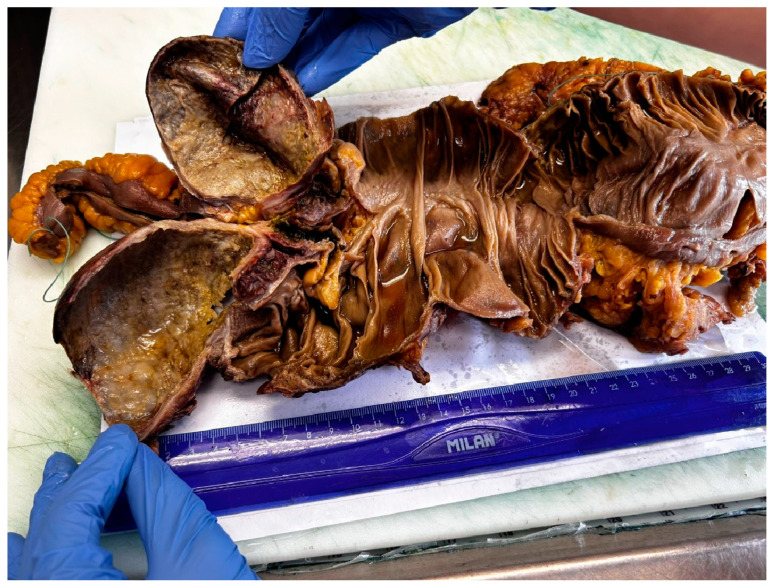
Macroscopic aspects of the harvested specimen.

The harvested specimen was fixed in 10% (*v*/*w*) neutral buffered formalin and sent to the pathology department for histopathologic examination. On macroscopic examination, the appendix was markedly dilated, with a diameter of 7 cm and a length of 11 cm, with intact gray and smooth serosa. The mucosa was irregular, gray-brown, and the wall was 0.8 cm thick. The cecum was slightly dilated, with a thin wall and a pale mucosa, with the folds partially erased (Figure 3). Five lymph nodules with a diameter between 0.2 and 0.7 cm were identified in the adipose tissue.

**Figure 4:**
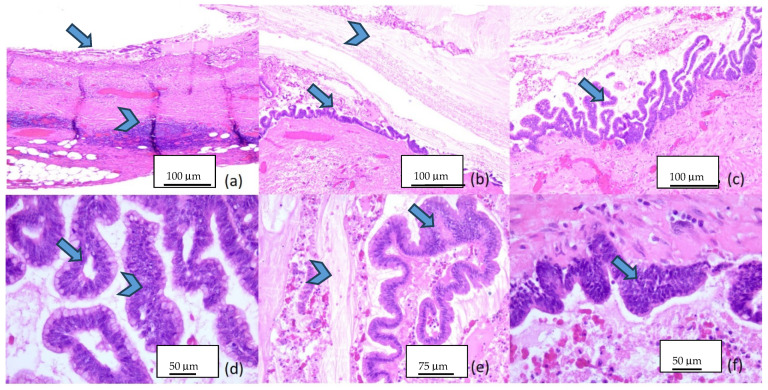
Microscopic aspects of the appendicular mucinous neoplasm: (**a**) appendicular wall with mucinous proliferation (arrow) and atrophy of the lymphoid tissue (arrowhead), 5×; (**b**) flat and filiform pattern of the proliferation (arrow), with endoluminal extravasated mucin (arrowhead), 5×; (**c**) filiform and villous pattern of the proliferation (arrow), 5×; (**d**) complex filiform and glandular pattern, with both low-grade (arrow) and high-grade atypical epithelial dysplasia (arrowhead), 20×; (**e**) low-grade atypia (arrow) and extravasated mucin (arrowhead), 10×; (**f**) high-grade atypical epithelial dysplasia (arrow), 20×.

Serial four-micrometer-thick sections were performed and stained with morphological Hematoxylin-Eosin for histopathologic examination. The microscopic examination of the harvested piece revealed a mucinous proliferation with a filiform and villous pattern and pushing margins. The tumor cells were columnar and had apical cytoplasmic mucin and hyperchromic, elongated and pseudostratified nuclei. Small areas with micropapillary features and enlarged pleomorphic nuclei with few atypical mitotic figures were observed. The appendix had atrophy of the lymphoid tissue, crypt loss and effacement of muscularis mucosae. There were areas of fibrosis and hyalinization. The extravasated extracellular mucin dissected through the lamina propria of the mucosa, in the submucosa. No mucin extrusion was observed at the peritoneal surface or at the base of the appendix (Figure 4).

**Figure 5:**
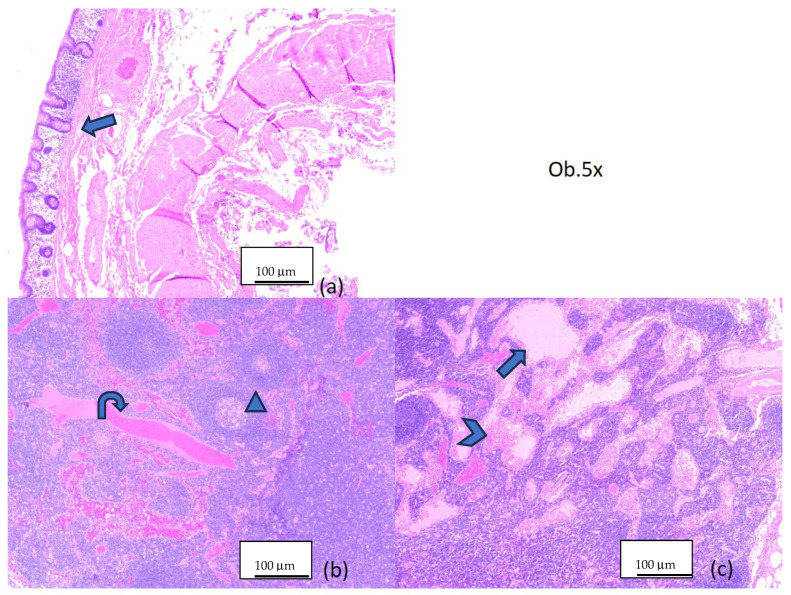
Microscopic aspects in Hematoxylin-Eosin staining, 5×: (**a**) colonic wall (arrow); (**b**,**c**) lymph node with catarrh (arrow), histiocytosis (arrowhead), vascular hyperemia (twisted arrow) and reactive follicular hyperplasia (triangle).

The cecum wall was distended by marked edema in the mucosa and submucosa (Figure 5a). There was no extravasated mucin in the lumen or on the serosa of the cecum. The five lymph nodules showed catarrh and sinus histiocytosis, as well as marked vascular hyperemia and reactive follicular hyperplasia (Figure 5b,c).

The morphological aspects in hematoxylin-eosin staining were sufficient to establish the histopathological diagnosis. The immunohistochemical study was not necessary.

This result confirmed that hemicolectomy was the right surgical decision in this case, because it ensures the necessary resection limits and, most probably, avoids a tumor recurrence.

The patient was referred to an Oncology Board and they decided that no oncological treatment was necessary for the time being. Imaging follow-up was recommended every 3 months in the first year after surgery.

## 3. Discussion

The mucocele of the appendix is a rare disease caused by accumulation of secreted mucus. The abnormal mucus secretion determines a gradual cystic dilatation of the appendix that can be initially asymptomatic and may result from the obstruction of the lumen, secondary to inflammation or neoplastic transformation of the appendix [11].

Women are more frequently affected by appendiceal mucocele than men, especially in the sixth decade of life; this higher frequency refers to appendiceal mucocele of any cause [12]. The case we presented is about a 68-year-old man and, since the occurrence of this disease in men is quite rare, it is worth reporting. Although gender prevalence is controversial in the literature, most studies present a male to female ratio of 1:4 [13,14,15,16].

In our case, the histopathological result showed both high-grade and low-grade mucinous neoplasm, associated with chronic inflammatory infiltration. As we already presented, mucinous neoplasms of the appendix are classically classified as low-grade mucinous neoplasm (LAMN), previously known as cystadenoma and high-grade mucinous neoplasm (HAMN), previously known as cystadenocarcinoma or as an intermediate stage between dysplasia and adenocarcinoma. Considering the simultaneous presence of both histopathological types in our case, we presume an evolutive transformation from low-grade dysplasia to high-grade dysplasia rather than the coexistence of two different pathologies.

A study including HAMNs [17] showed that nearly 50% of the patients had low-grade focal or segmental appendiceal involvement, suggesting a process of progression from LAMN to HAMN. In the patients with diffuse high-grade dysplasia, the de novo occurrence of this disease cannot be excluded. Microscopically, high-grade dysplasia presents similar changes in the appendiceal wall as low-grade dysplasia and the cytologic atypia seen in the epithelium is of higher grade than that seen in low-grade dysplasia. Based on the mutational analysis and the morphologic evidence, LAMN and HAMN are thought to have a common histogenesis, and it is likely that HAMN derives from LAMN [18].

When it comes to colorectal cancer, some studies have provided evidence on the progression from low-grade dysplasia to high grade dysplasia and up to 50% of the studied patients with low grade dysplasia developed more advanced lesions [19]. Furthermore, this transformation seems to be favorized by some circumstances. Chronic inflammation is the primary risk factor that can lead to low-grade dysplasia, followed by high-grade dysplasia and sometimes by adenocarcinoma [20,21]. Considering this sequence of events, a history of low-grade dysplasia is a major risk factor for developing advanced neoplasia in the presence of certain factors [22,23]. Independent risk factors for the progression to advanced neoplasia after detection of low-grade dysplasia are an age above 55 years and male gender. In the case of the presence of several risk factors, the incidence rate of advanced neoplasia at 10 years after detecting low-grade dysplasia increased from 8% to 20% [24].

Patients with inflammatory bowel disease are infrequently diagnosed with appendicular neoplasms and it is suggested that the inflammatory nature of the disease can involve the appendiceal orifice [25]. A higher incidence rate of appendiceal mucocele, particularly cystadenoma, was observed in patients with chronic inflammation. The obstruction of the appendiceal orifice plays a role in the development of appendiceal mucocele, and the blockage might be due to inflammation [25].

Knowing the fact that the structure of both the appendix and the colon is almost identical and considering the results of the studies, we can expect that chronic inflammation, consisting of lymphocytes, macrophages, plasmocytes and the presence of both histopathological types in our case are an evidence of the evolution from low-grade dysplasia to high-grade dysplasia in the presence of cumulative risk factors in our patient: increased age (68 years), male gender and chronic inflammatory infiltration. We also take into consideration the possibility of coexistence of both pathologies, but it is unlikely for two histopathological types to arise de novo on the same site.

Appendiceal mucinous neoplasms are asymptomatic in over 50% of the cases and in about 30% of symptomatic patients, the clinical expression is of acute appendicitis. This condition may be associated with other colonic neoplasia. The main symptoms are right lower quadrant pain or non-specific abdominal pain, nausea, vomiting, features of intestinal obstruction from intussusception, gastrointestinal bleeding, fever, anorexia, weight loss and fatigue. Physical examination can reveal right lower quadrant tenderness with localized guarding, a palpable mass and, in the case of peritoneal invasion with mucoid cells, presence of ascites [5,26,27].

Our patient, in his seventh decade, presented with the main clinical symptoms of acute appendicitis, which may indicate the malignant character of the appendiceal mucocele according to some published studies, whereas benign mucoceles are usually asymptomatic. The symptoms might also have been enhanced by the appendiceal inflammation confirmed by the CT examination and the histopathological result that revealed inflammatory changes adjacent to vascular structures from the muscularis propria.

Considering the fact that mucoceles, either neoplastic or non-neoplastic, are asymptomatic in over 50% of cases, imaging plays an important role in diagnosis and management. Ultrasonography can reveal elongated or cystic lesions in the right lower quadrant with an internal pathognomonic “onionskin” appearance representing lamellated layers of mucin. Calcification of the appendiceal wall creates echogenicity near the wall and distal acoustical shadowing. Abdominal X-ray examination is not very useful, but it may show calcifications in the right lower quadrant [26,28]. When patients present with acute appendicitis-like symptoms, the CT examination can show an appendix that is longer than 15 mm and presents thickened or irregular walls, filled with homogenous, low-attenuation content, abnormalities along the cecal contour or intraluminal smooth lesions at the expected position of the appendiceal orifice, all characteristics of mucous neoplasia. Appendicitis secondary to neoplasia presents as a dilated appendix with a thickened, hyperemic wall, and surrounding inflammatory changes, often without signs of an underlying neoplastic process [6,8,26,28]. The interface of the mucinous nodules or the fibrous tissue between them can be seen as septations [29]. When magnetic resonance imaging (MRI) is performed, intraluminal and peri-appendiceal mucin appears bright on T2-weighted images and the intensity of the signal in T1 weighted images depends on the mucin concentration [30]. For an accurate diagnosis, carcinoembryonic antigen can be tested. Elevated levels are often seen in mucinous cystadenocarcinoma, but are rare in the case of mucinous cystadenoma [31,32].

The abdominal CT performed in our patient revealed a cystic dilated appendix, with a length of 130 mm and a diameter of 75 mm, situated behind the cecum, with an ascending position, a thickened implantation base and a thickened cecal base with peri-appendicular inflammatory changes extended to the right paracolic recess.

The treatment of the mucoceles is surgical and depends on its size and histological type and the involvement of other structures. The main options are appendicectomy or right hemicolectomy. Appendicectomy is recommended for the small-diameter and benign mucoceles, for example, retention cysts. In this situation, appendicectomy requires a wide mesoappendix resection and a careful dissection to avoid perforation and peritoneal contamination. In non-perforated benign mucoceles, the simple appendicectomy is curative [33,34,35]. Right hemicolectomy is performed if a malignancy is suspected, in case of mucinous cystadenocarcinoma, if the appendiceal base or other adjacent structures like the cecum and lymph nodes are involved and if the diameter of the mucocele is significantly enlarged. In open laparotomy, careful exploration of the entire peritoneal cavity and other viscera like the colon is possible, considering the potential association of an appendiceal mucocele and colonic neoplasia. Open laparotomy with right hemicolectomy is the best option if the appendiceal mucocele is large, because of the difficult dissection. It is important to prevent the rupture of the appendix to avoid intraperitoneal dissemination of mucin known as pseudomyxoma peritonei [33,34,36,37,38].

In our case, after analyzing all the available data, we decided that the best option is to perform an open laparotomy, for the above-mentioned reasons: the size of the appendiceal mucocele (13/7.5/7 cm), the ascending retrocecal position associated with the peri-appendiceal and pericecal inflammatory process, both causing a difficult dissection and a high suspicion of malignancy. Open laparotomy offered a proper view of the lesion and allowed us to perform a proper exploration of the entire peritoneal cavity. The mucocele had an ascending retrocecal position, was adherent to the cecum and the right colon, having a length of 13 cm and a diameter of 7.5 cm. The mucosa had a brown-gray color. The cystic transformed appendix and the cecum presented a large communication through the appendicular orifice. Both structures appeared with thick, fibrous walls and inflammatory changes. No other macroscopic lesions were found in the peritoneal cavity. Considering all the facts, including the possibility of a malignant etiology, right hemicolectomy with side-to-side ileo-colonic anastomosis was performed.

Comparing our case with other recently published high-grade dysplasia appendiceal mucocele, we found some interesting aspects that are worth mentioning. The presentation of the patients can be different, from mimicking an acute appendicitis [39] to a long history of pelvic pain [17], upper abdominal pain [40], non-specific abdominal symptoms like unintentional weight loss, decreased appetite or constipation [41] or simply an accidental finding. The dimensions of an appendiceal mucocele may become impressive, of up to 44 cm, as is the case published by Lu et al. [41]. The therapeutic approach can be extremely different, ranging from appendectomy to ileo-colonic resections or right colectomy, depending on surgeon’s experience, intraoperative findings or extemporaneous examination [39]. Imaging is essential for the diagnosis of appendiceal mucocele, avoiding a possible intraoperative surprise and becoming a gold standard for this pathology. The histopathological examination remains of paramount importance for the subsequent management of those patients, the complete examination of the resected specimen being mandatory [39] for a correct and complete diagnosis.

Although recent studies showed no risk of recurrence or progression to pseudomyxoma peritonei in mucinous neoplasms confined to the appendix, even in the case of high-grade dysplasia [42], we believe that further studies are needed, using larger groups of patients, in order to find the best therapeutic approach and to provide the best possible outcome for these patients. Due to the relatively new and ambiguous terminology used to define appendiceal mucinous neoplasm and the lack of a large series of patients with this pathology, we consider it crucial to evaluate and follow up every case of appendiceal mucocele, especially if dysplasia is involved, in order to find the best therapy for these patients and to provide a good outcome, with no recurrence or progression.

## 4. Conclusions

Appendiceal mucocele is an interesting condition that could have a simple explanation as the obstruction of the appendiceal orifice or it could be an association of factors and local phenomena that lead to a complex pathology. The fact that the microscopic aspect is similar to the one found in colorectal tumors is a reason to implement a more aggressive therapeutic algorithm and to approach this pathology like a potential malignancy. As it is recommended in colorectal neoplasms, the neoplastic appendiceal mucocele need surgical treatment in concordance with the grade of dysplasia.

## Data Availability

The original contributions presented in the study are included in the article, further inquiries can be directed to the corresponding author.

## References

[1] Louis T.H., Felter D.F. (2014). Mucocele of the Appendix. Proc. (Bayl. Univ. Med. Cent.).

[2] Spyropoulos C., Rentis A., Alexaki E., Triantafillidis J.K., Vagianos C. (2014). Appendiceal mucocele and pseudomyxoma peritonei; the clinical boundariesof a subtle disease. Am. J. Case Rep..

[3] Higa E., Rosai J., Pizzimbono C.A., Wise L. (1973). Mucosal hyperplasia, mucinous cystadenoma, and mucinous cystadenocarcinoma of the appendix. A re-evaluation of appendiceal “mucocele”. Cancer.

[4] Sugarbaker P.H., Bland K.I., Büchler M.W., Csendes A., Sarr M.G., Garden O.J., Wong J. (2009). Appendiceal Epithelial Neoplasms and Pseudomyxoma Peritonei, a Distinct Clinical Entity with Distinct Treatments. General Surgery. Principles and International Practice.

[5] Abuoğlu H., Yıldız M.K., Kaya B., Odabaşı M. (2017). Clinicopathological analysis of patients operated for appendiceal mucocele. Ulus. Travma Acil Cerrahi Derg..

[6] Santos S.F., Horta M., Rosa F., Rito M., Cunha T.M. (2022). Mucocele of the appendix: What to expect. Radiol. Bras..

[7] Hoehn R.S., Rieser C.J., Choudry M.H., Melnitchouk N., Hechtman J., Bahary N. (2021). Current Management of Appendiceal Neoplasms. Am. Soc. Clin. Oncol. Educ. Book.

[8] Spanos C.P., Kaiser A.M., Steele S.R., Hull T.L., Read T.E., Saclarides T.J., Senagore A.J., Whitlow C.B. (2016). Appendiceal Neoplasms. The ASCRS Textbook of Colon and Rectal Surgery.

[9] Hatch Q.M., Gilbert E.W. (2018). Appendiceal Neoplasms. Clin. Colon Rectal Surg..

[10] Umetsu S.E., Kakar S. (2023). Staging of appendiceal mucinous neoplasms: Challenges and recent updates. Hum. Pathol..

[11] BB S.K., Jasuja P. (2019). Appendiceal mucocele—A rare case report. Int. J. Surg. Case Rep..

[12] Gundogar O., Kimiloglu E., Komut N., Cin M., Bektas S., Gonullu D., Ilgun A.S., Erdogan N. (2018). Evaluation of appendiceal mucinous neoplasms with a new classification system and literature review. Turk. J. Gastroenterol..

[13] Aho A.J., Heinonen R., Laurén P. (1973). Benign and malignant mucocele of the appendix. Acta Chir. Scand..

[14] Wang H., Chen Y.Q., Wei R., Wang Q.B., Song B., Wang C.Y., Zhang B. (2013). Appendix Mucocele: A Diagnostic Dilemma in Differentiating Malignant from Benign Lesions with CT. Am. J. Roentgenol..

[15] Pitiakoudis M., Argyropoulou P.I., Tsaroucha A.K., Prassopoulos P., Simopoulos C. (2003). Cystadenocarcinoma of the appendix: An incidental imaging finding in a patient with adenocarcinomas of the ascending and the sigmoid colon. BMC Gastroenterol..

[16] Coulibaly A., Simaga A.K., Sissoko M., Koumaré S., Konaré S., Doumbia M., Sanogo Z.Z. (2022). Appendicular Mucocele: About a Case Observed in Bamako. Surg. Sci..

[17] Gonzalez R.S., Carr N.J., Liao H., Pai R.K., Agostini-Vulaj D., Misdraji J. (2022). High-Grade Appendiceal Mucinous Neoplasm: Clinicopathologic Findings in 35 Cases. Arch. Pathol. Lab. Med..

[18] Liao X., Vavinskaya V., Sun K., Hao Y., Li X., Valasek M., Xu R., Polydorides A.D., Houldsworth J., Harpaz N. (2020). Mutation profile of high-grade appendiceal mucinous neoplasm. Histopathology.

[19] Rutter M.D., Saunders B.P., Wilkinson K.H., Rumbles S., Schofield G., Kamm M.A., Williams C.B., Price A.B., Talbot I.C., Forbes A. (2006). Thirty-year analysis of a colonoscopic surveillance program for neoplasia in ulcerative colitis. Gastroenterology.

[20] Beaugerie L., Itzkowitz S.H. (2015). Cancers complicating inflammatory bowel disease. N. Engl. J. Med..

[21] Kim E.R., Chang D.K. (2014). Colorectal cancer in inflammatory bowel disease: The risk, pathogenesis, prevention and diagnosis. World J. Gastroenterol..

[22] Fumery M., Dulai P.S., Gupta S., Prokop L.J., Ramamoorthy S., Sandborn W.J., Singh S. (2017). Incidence, risk factors, and outcomes of colorectal cancer in patients with ulcerative colitis with low-grade dysplasia: A systematic review and meta-analysis. Clin. Gastroenterol. Hepatol..

[23] Wu X.-R., Zheng X.-B., Huang Y., Cao Q., Zhang H.-J., Miao Y.-L., Zou K.-F., Chen M., Zhang F.-M., Mei Q. (2019). Risk factors for colorectal neoplasia in patients with underlying inflammatory bowel disease: A multicenter study. Gastroenterol. Rep..

[24] Choi C.-H.R., Ignjatovic-Wilson A., Askari A., Lee G.H., Warusavitarne J., Moorghen M., Thomas-Gibson S., Saunders B.P., Rutter M.D., Graham T.A. (2015). Low-grade dysplasia in ulcerative colitis: Risk factors for developing high-grade dysplasia or colorectal cancer. Am. J. Gastroenterol..

[25] Lakatos P.L., Gyori G., Halasz J., Fuszek P., Papp J., Jaray B., Lukovich P., Lakatos L. (2005). Mucocele of the appendix: An unusual cause of lower abdominal pain in a patient with ulcerative colitis-. A case report and review of literature. World J. Gastroenterol..

[26] Tirumani S.H., Fraser-Hill M., Auer R., Shabana W., Walsh C., Lee F., Ryan J.G. (2013). Mucinous neoplasms of the appendix: A current comprehensive clinicopathologic and imaging review. Cancer Imaging.

[27] Carr N., Sobin L., Bosman F.T., Carneiro F., Hruban R.H., Theise N.D. (2010). Tumors of the appendix. In: WHO Classification of tumours of the digestive system. World Health Organization Classification of Tumours.

[28] Dachman A., Lichtenstein J., Friedman A.C. (1985). Mucocele of the appendix and pseudomyxoma peritonei. Am. J. Roentgenol..

[29] Sulkin T.V.C., O’Neill H., Amin A.I., Moran B. (2002). CT in pseudomyxoma peritonei: A review of 17 cases. Clin. Radiol..

[30] Pickhardt P.J., Levy A.D., Rohrmann C.A., Kende A.I. (2003). Primary neoplasms of the appendix: Radiologic spectrum of disease with pathologic correlation. Radiographics.

[31] McFarlane M.E., Plummer J.M., Bonadie K. (2013). Mucinous cystadenoma of the appendix presenting with an elevated carcinoembryonic antigen (CEA):Report of two cases and review of the literature. Int. J. Surg. Case Rep..

[32] Saftescu S., Popovici D., Opean C., Negru A., Croitoru A., Zemba M., Yasar I., Preda M., Negru S. (2020). Endurance of erythrocyte series in chemotherapy. Exp. Ther. Med..

[33] Miraliakbari R., Chapman W.H. (1999). Laparoscopic treatment of an appendiceal mucocele. J. Laparoendosc. Adv. Surg. Tech..

[34] Dhage-Ivatury S., Sugarbaker P.H. (2006). Update on the surgical approach to mucocele of the appendix. J. Am. Coll. Surg..

[35] Kılıç M.Ö., İnan A., Bozer M. (2014). Four mucinous cystadenoma of the appendix treated by different approaches. Ulus. Cerrahi Derg..

[36] Stocchi L., Wolff B.G., Larson D.R., Harrington J.R. (2003). Surgical treatment of appendiceal mucocele. Arch. Surg..

[37] Persaud T., Swan N., Torreggiani W.C. (2007). Giant mucinous cystadenoma of the appendix. Radiographics.

[38] Carr N.J., McCarthy W.F., Sobin L.H. (1995). Epithelial noncarcinoid tumors and tumor-like lesions of the appendix. A clinicopathologic study of 184 patients with a multivariate analysis of prognostic factors. Cancer.

[39] Cestino L., Festa F., Cavouti G., Bonatti L., Soncini S., Dani L., Quaglino F. (2020). Appendiceal mucocele: Three cases with different presentation and review of the literature. J. Surg. Case Rep..

[40] Kearsey C.C., Dristas S., Mathur M., Wild J. (2024). “It’s just a mucocele”: Case report of a massive appendiceal mucocele presenting as a left upper quadrant mass. Ann. R. Coll. Surg. Eng..

[41] Lu A., Cho J., Vazmitzel M., Layfield L., Staveley-O’Carroll K., Gaballah A., Rao D. (2021). High-grade appendiceal mucinous neoplasm presenting as a giant appendiceal mucocele. Radiol. Case Rep..

[42] Polydorides A.D., Wen X. (2022). Clinicopathologic parameters and outcomes of mucinous neoplasms confined to the appendix: A benign entity with excellent prognosis. Mod. Pathol..

